# Hydraulic performance prediction and optimization of an engine cooling water pump using computational fluid dynamic analysis

**DOI:** 10.1371/journal.pone.0253309

**Published:** 2021-06-15

**Authors:** Libin Tan, Yuejin Yuan, Man Zhang

**Affiliations:** College of Mechanical and Electrical Engineering, Shaanxi University of Science and Technology, Xi’an, Shaanxi, China; Tianjin University, CHINA

## Abstract

In current research, the hydraulic performance prediction and optimization of an engine cooling water pump was conducted by computational fluid dynamic (CFD) analysis. Through CFD simulation, the pump head, shaft power and efficiency for the original pump at volume flow rate 25 L/min and impeller rotating speed 4231 r/min were 3.87 m, 66.7 W and 23.09% respectively. For improving hydraulic performance, an optimization study was carried out. After optimization, four potential optimized designs were put forward. The efficiency of the optimized design No.1 for engine cooling water pump was nearly 6% higher than that of the original pump model; and the head of the optimized design No.2 for engine cooling water pump was 9% higher than that of the original pump model. Under the condition of maintaining the pump head and considering comprehensive improvement effect, the optimized design No.3 was considered as the best design and selected as the test case for validating the optimum design. The hydraulic performance predictions for this optimum engine cooling water pump agreed well with experimental data at design condition with relative discrepancies of 2.9% and 5.5% for the pump head and pump efficiency, respectively. It proved that performance prediction calculation model and the automatic optimization model were effective. This research work can provide theoretical basis for the design, development and optimization of engine cooling water pump.

## 1. Introduction

As the core power source of the cooling system of the water-cooled engine, the cooling function of the cooling water pump was mainly to use the high-speed rotating impeller to drive the coolant circulation flow to realize the energy conversion, reduce the heat brought by the engine operation, and avoid the engine overheating and running failure. It can be seen that the performance of water pump directly affects the working performance and service life of the engine [1]. The design and development of cooling water pump must meet the technical quality indexes such as hydraulic performance, vibration noise and fatigue strength, so as to produce the pump products with competitive advantage [2]. However, in useful undertakings, design engineers usually designed engine cooling water pumps according to theory or experience, and the preliminary design of water pumps often fails to have meeting with the expected hydraulic performance. Traditional methods for pump design and optimization mainly adopted the trial-and-error strategy by manufacturing and testing prototype pumps, which were high in price and time getting used up. As an outcome of that, it was important to carry out the parametric modeling design on the basic structure model and the parameter optimization research on the core components.

Nowadays, to overcome the limitations of experimental cost and labor consumption problems, Computational fluid dynamics (CFD) methodology has been widely used for predicting hydraulic performance of centrifugal pumps and considered as a powerful tool for the optimal design of pumps [3–5]. In the process of pump operation, the fluid flow caused by the rotation of internal impeller was complicated. As an important flow passage component of pump, the hydraulic parameters of impeller will have an important impact on the performance of pump. Nowadays, many researchers had conducted the numerical simulation analysis on performance characteristics of pumps. For example, Anagnostopoulos [6] investigated the hydraulic performance curves of centrifugal pump and found the optimal geometry shape of impeller blade that could maximize the pump efficiency among a set of blade angles. Ding et al [7]. discovered that changing the blade exit angle has a greater impact on the efficiency of the centrifugal pump and less impact on the head. Saeed et al [8]. analyzed the effects of geometrical and operational uncertainties on the hydraulic performance of a low specific-speed centrifugal pump, and observed that the variation of the pump head coefficient under operational and geometrical uncertainties was significant, the pump efficiency showed a robust behavior under assumed uncertain conditions. Zhang et al [9]. analyzed the influence of different blade angles on centrifugal pump performance. Lin et al [10]. had emulated the inner flow field of the centrifugal pump by CFD and optimized the flow field characteristics by controlling the impellers cutting process for reducing noise and isolating vibration. Wang et al [11]. had analyzed the centrifugal pump with five different geometrical parameters for improving performance of marine centrifugal pump. Zhao et al. [12] proposed a multi-objective optimization of a low specific speed centrifugal pump with two objectives of the maximum hydraulic efficiency and the minimum static head difference. Li et al. [13] completed the optimized designs of engine cooling water pump(ECWP) and analyzed the internal flow patterns of those pumps by ANSYS CFX, showing that pump head had a little drop when the blade number reduced and the results derived from simulation data have been proven to be reliable. The CFD simulation is proved to be an essential tool for analyzing the internal airflow patterns and velocity profiles, which benefits engineer and manufacturers to improve the design of existing product [14, 15].

A number of researchers investigated the influence of various parameters on hydraulic performance of centrifugal pump and seek proper methodology for obtaining an optimized centrifugal pump with high hydraulic performance. However, most of these studies were based on the single optimization objective optimization or the comparative analysis of multiple scheme simulation results for finding the optimal structure. There was an empirical attempt to design and optimize based on the above methods, which may cause the delay risk of product design and development cycle time. If the pump design can be optimized automatically, the delay risk can be decreased. However, at present, there were relatively few researches on "prototype model- numerical simulation-optimization" integrated optimization analysis for automatic optimization. Therefore, it was quite meaningful for finding a way to achieve this integrated optimization analysis through the integration with parametric model, theoretical prediction, and engineering optimization.

In this research work, an engine cooling water pump was taken as the research object, and the performance evaluation of original engine cooling water pump was investigated. The objective of this work was to improve the pump head, efficiency and reduce the shaft power of engine cooling water pump for obtaining an optimization design of engine cooling water pump. In order to achieve the optimization quickly, an automatic optimization model was built by integrating the 3D parametric model construction software CATIA with fluid dynamics simulation software STAR-CCM+. Through the automatic update of parametric impeller geometry model and the continuous iteration analysis of pump performance evaluation, the integrated optimization method of "parametric model-numerical simulation" was fully succeeded to realize automatic optimization of pump performance, automatic output and storage of calculation results, and put forward corresponding optimization designs to improve the working performance of pump. The research results can lay a theoretical foundation for the design, development and performance optimization of engine cooling water pump.

## 2. CFD modelling of engine cooling water pump

### 2.1 Physical model

**Fig 1:**
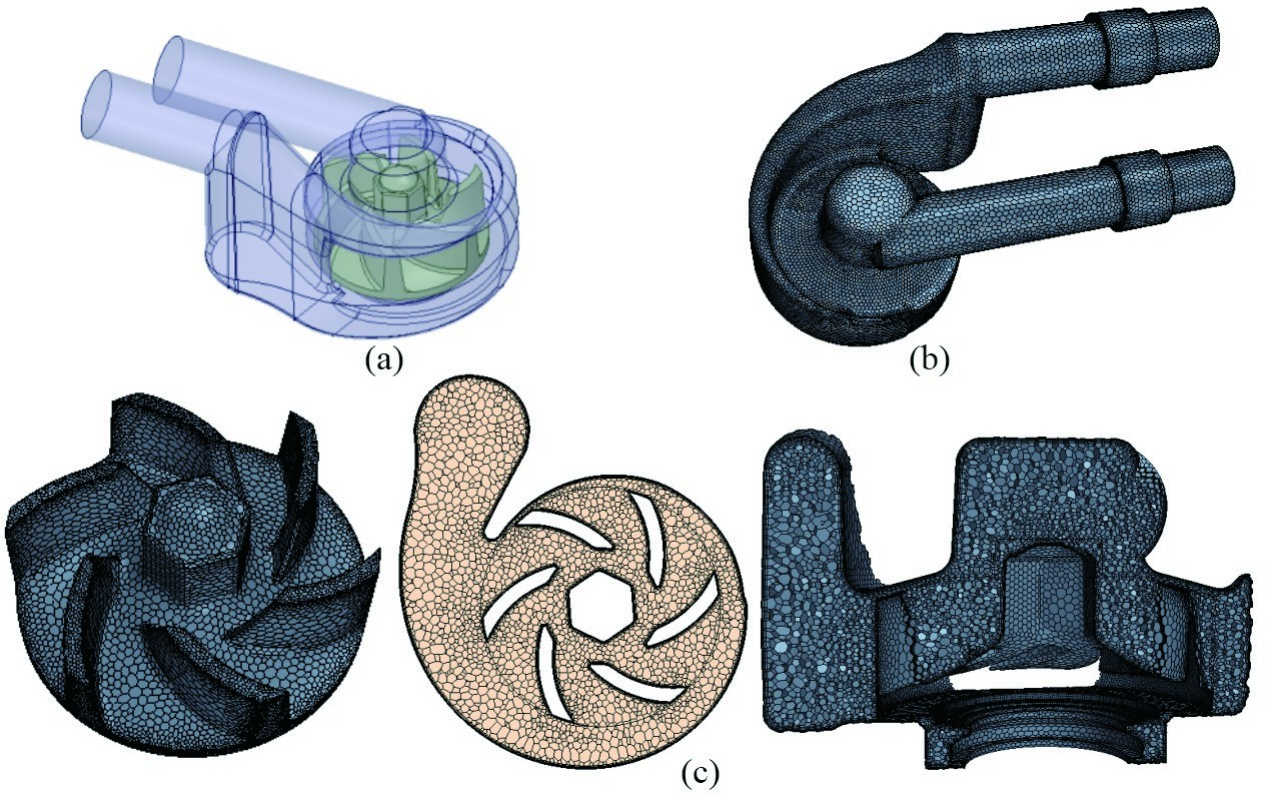
Computational region for engine cooling water pump: (a) Geometry model; (b) Mesh model; (c) Mesh details.

In this research, an engine cooling water pump was investigated, which design specific speed ($n_{sd}=3.65n_{d}\sqrt{Q_{d}}/H_{d}{}^{0.75}$) at design volume flow rate mode is 115, where *n*_*d*_ was the rotational speed of the impeller with unit (r/min), *Q*_*d*_ was the designed volume flow rate with unit (m^3^/s), and *H* is the designed head at designed volume flow rate with unit (m). The three dimensional geometric model, mesh model and mesh details of the engine cooling water pump were shown in Fig 1. The geometric model of engine cooling water pump includes the impeller and volute part. The mesh generation was an important step in the process of numerical simulation by directly affecting the accuracy and calculation time. The computational fluid domain model of pump was imported into CFD analysis software STAR-CCM+ 11.06 for grid generation, boundary setting, internal flow field pattern analysis and results post-processing. The polyhedron grid technology and prism layer grid technology available in STAR-CCM+ were selected to generate the computational grid of the pump, and the feature line encryption method was adopted to encrypt the impeller tip. In order to fully develop the turbulence and reduce the back-flow, a 100 mm stretch layer grid was used in the numerical simulation software. For excluding the influence of grid number on calculation accuracy of pump hydraulic performance, three mesh models (Coarse mesh model, Middle mesh model and Fined mesh model) with different grid numbers are generated. Taking the design point as an example, the pump head and the efficiency of the pump are calculated and the results are shown in Table 1. It can be observed that all the errors of the pump heads and the efficiencies by those mesh models are less than 5% and 10%, respectively. It should be noted that the calculated flow efficiency and pump head are slightly higher than the experimental data. The over-prediction may be attributed to the neglecting of the leakage loss induced by the surface roughness, the clearances in pumps, and the mechanical loss caused by mechanical seals and bearings. To gain accurate results and consider the computational cost, the middle mseh model is chosen for conducting the following hydraulic performance of centrifugal pump with the grid number about 2.1 million. Grid independence tests proved this grid can give a grid independent solution and can obtain a good prediction accuracy.

**Table 1:** Pump head and pump efficiency calculated by different mesh models.

Mesh model	Grid number	Head(m)			Efficiency(%)		
		CFD	Exp	error	CFD	Exp	error
Coarse	1153225	3.902	3.72	2.80%	23.94	21.8	9.80%
Middle	2107555	3.874		4.20%	23.09		5.90%
Fine	3586885	3.871		4.10%	23.06		5.80%

### 2.2 Mathematical model

Fluid flow satisfied three conservation laws: mass conservation law, momentum conservation law and energy conservation law [16]. When the flow was turbulent, the whole system must follow the turbulent transport equation. The mathematical descriptions of these conservation laws were collectively referred to as control equations. In this paper, the k-ε turbulence model provided in STAR-CCM+ was used for numerical calculation and the wall function method was set by Two-Layer All Y+ Wall Treatment recommended in STAR-CCM+ [17]. It should be noted that the local area near the wall was densified by prism mesh layers. It aims to reflect the wall boundary layer flow. The y+ was kept in an appropriate range for the turbulence model and wall treatment [18]. It was assumed that the flow in engine cooling water pump was incomprehensible and steady and the influence of liquid temperature was not considered in this work. Therefore, the governing equations for fluid flow (continuity equation and momentum equation) and transport equations for k-ε turbulence model equations were listed as follows [19, 20]:

1. Continuity equation
∂u/∂x+∂v/∂y+∂w/∂z=0 (1)
2. Momentum equation (N-S equation)
$$\begin{array}{l} \rho \partial u/\partial t+\rho div(uU)=-\partial p/\partial x+\partial \tau_{xx}/\partial x+\partial \tau_{yx}/\partial y+\partial \tau_{zx}/\partial z\\ \rho \partial v/\partial t+\rho div(vU)=-\partial p/\partial y+\partial \tau_{xy}/\partial x+\partial \tau_{yy}/\partial y+\partial \tau_{zy}/\partial z\\ \rho \partial w/\partial t+\rho div(wU)=-\partial p/\partial z+\partial \tau_{xz}/\partial x+\partial \tau_{yz}/\partial y+\partial \tau_{zz}/\partial z \end{array}$$ (2)
  where *ρ* was the density, kg/m^3^; *u*, *v*, *w* were the velocity components, m/s; *x*, *y*, *z* were spatial coordinates, m; *U* was the velocity vector, *p* was the pressure, Pa; *τ_xy_, τ_xx_, τ_xz_*, et al were the components of viscous stress *τ*, Pa.
3. Transport equations for k-ε turbulence model
$$\begin{array}{l} \rho \frac{\partial (k)}{\partial t}+\rho u_{i}\frac{\partial (k)}{\partial x_{i}}=\frac{\partial }{\partial x_{j}}[(\mu +\frac{\mu_{t}}{\sigma_{k}})\frac{\partial k}{\partial x_{j}}]+\\ G_{k}+G_{b}-\rho \varepsilon -Y_{M}+S_{k}\\ \rho \frac{\partial (\varepsilon )}{\partial t}+\rho u_{i}\frac{\partial (k)}{\partial x_{i}}=\frac{\partial }{\partial x_{j}}[(\mu +\frac{\mu_{t}}{\sigma_{\varepsilon }})\frac{\partial \varepsilon }{\partial x_{j}}]+\\ C_{1\varepsilon }\frac{\varepsilon }{k}(G_{k}+C_{3\varepsilon }G_{b})-C_{2\varepsilon }\rho \frac{\varepsilon^{2}}{k}+S_{\varepsilon } \end{array}$$ (3)
  where *k* was the turbulent kinetic energy, m^2^/s^2^; *ε* was the turbulent dissipation rate, m^2^/s^3^; *μ_t_* was the turbulent viscosity, N.s/m^2^; *G_k_* was the turbulent kinetic energy term generated by the velocity gradient, *G_b_* was the turbulent kinetic energy term generated by the buoyancy, *Y_M_* was the pulsating expansion term, *C*_1*ε*_, *C*_2*ε*_, *C*_3*ε*_ were empirical constants, *σ_k_, σ_ε_* were the Prandtl number corresponding to the turbulent kinetic energy and the dissipation rate respectively; *S*_*k*_ and *S*_*ε*_ were user-defined source terms.

### 2.3 Boundary conditions

As for the CFD analysis method for rotating parts in CFD simulation software, numerical methods such as frozen rotor approach, mixing plane, sliding mesh, dynamic mesh, moving reference frame method and some others were often used [21]. Among them, sliding mesh and dynamic mesh were used for transient calculation. Frozen rotor approach, mixed plane and moving reference method were used for steady calculation. In STAR-CCM+, the impeller rotation in steady state is accomplished by a moving reference frame (MRF) modeling technique [22]. In MRF approach, a separate region enclosing the entire rotation region must be defined, and a rotating reference frame was assigned to that region [23]. Therefore, a constant grid flux will be generated in the appropriate conservation equations. Therefore, in this work, Moving Reference Frame method was used to modeling rotation of the impeller. The impeller rotation speed of the engine cooling water pump was 4231 r/min. The boundary condition for pump inlet was set as the stagnation inlet with relative pressure 0 Pa, and boundary condition for outlet was set as the mass flow outlet boundary. The fluid medium was pure water at 80°C, with a density of 978 kg / m^3^. The rest of the wall boundary was considered as slip free wall boundary.

## 3. Flow field analysis results and discussion

### 3.1 Pump performance

**Fig 2:**
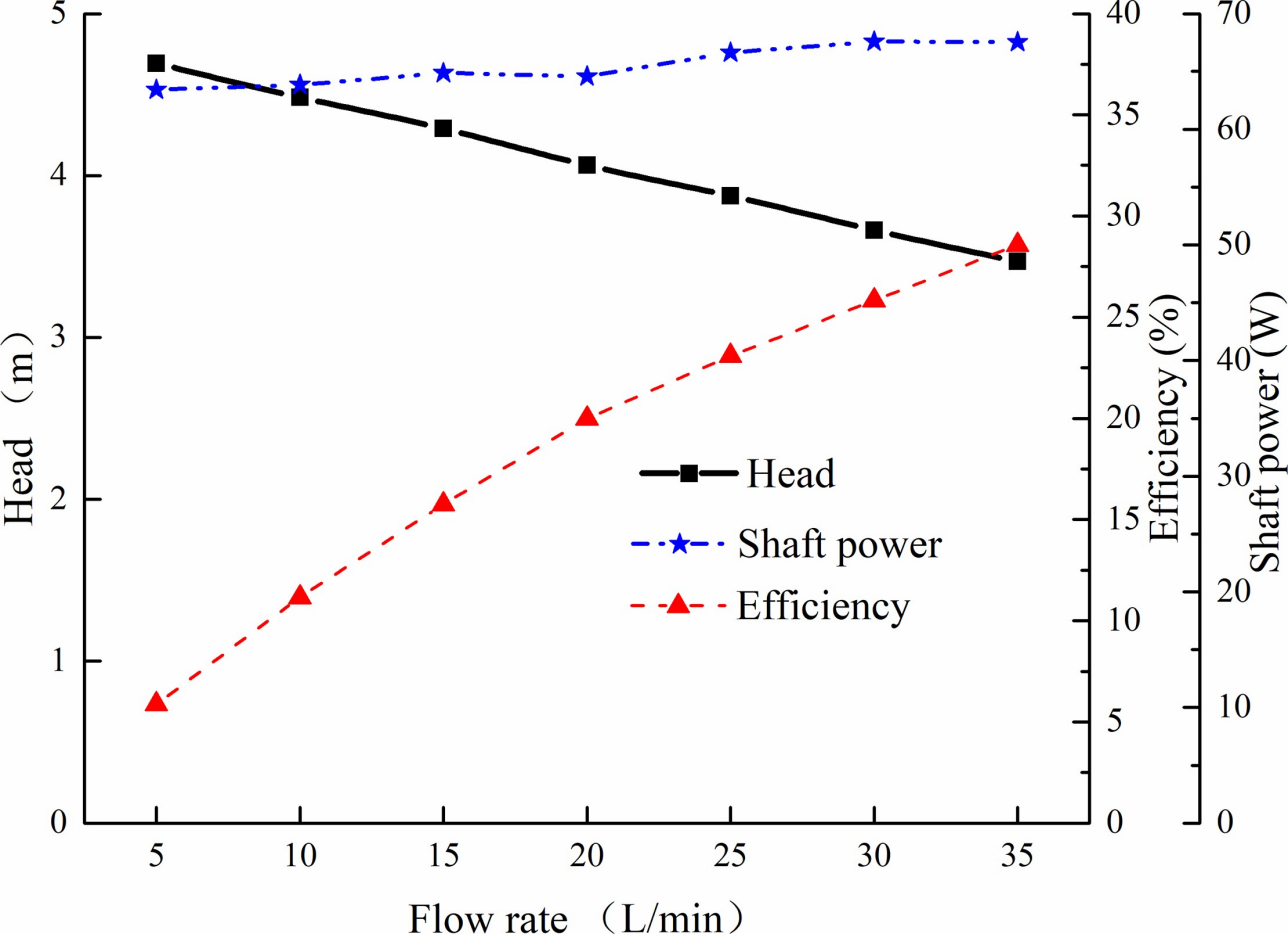
Hydraulic performance cures for original engine cooling water pump.

Fig 2 showed the pump hydraulic performance comparison curves for original cooling water pump. Through CFD simulation, the pump head, shaft power and efficiency for original pump at designed volume flow rate 25 L/min and impeller rotating speed 4231 r/min were 3.87 m, 66.7 W and 23.09%, respectively. Experimental validation (Seen in Section 4.4) proved that CFD methodology can be a feasible method for hydraulic performance prediction. The efficiency of original water pump at its design point was a little lower. Therefore, it was necessary to make a optimization study on improving its hydraulic performance for engine cooling water pump, assuring that it can meet the cooling requirements when the pump was applied in engine cooling system.

### 3.2 Internal flow field of pump

**Fig 3:**
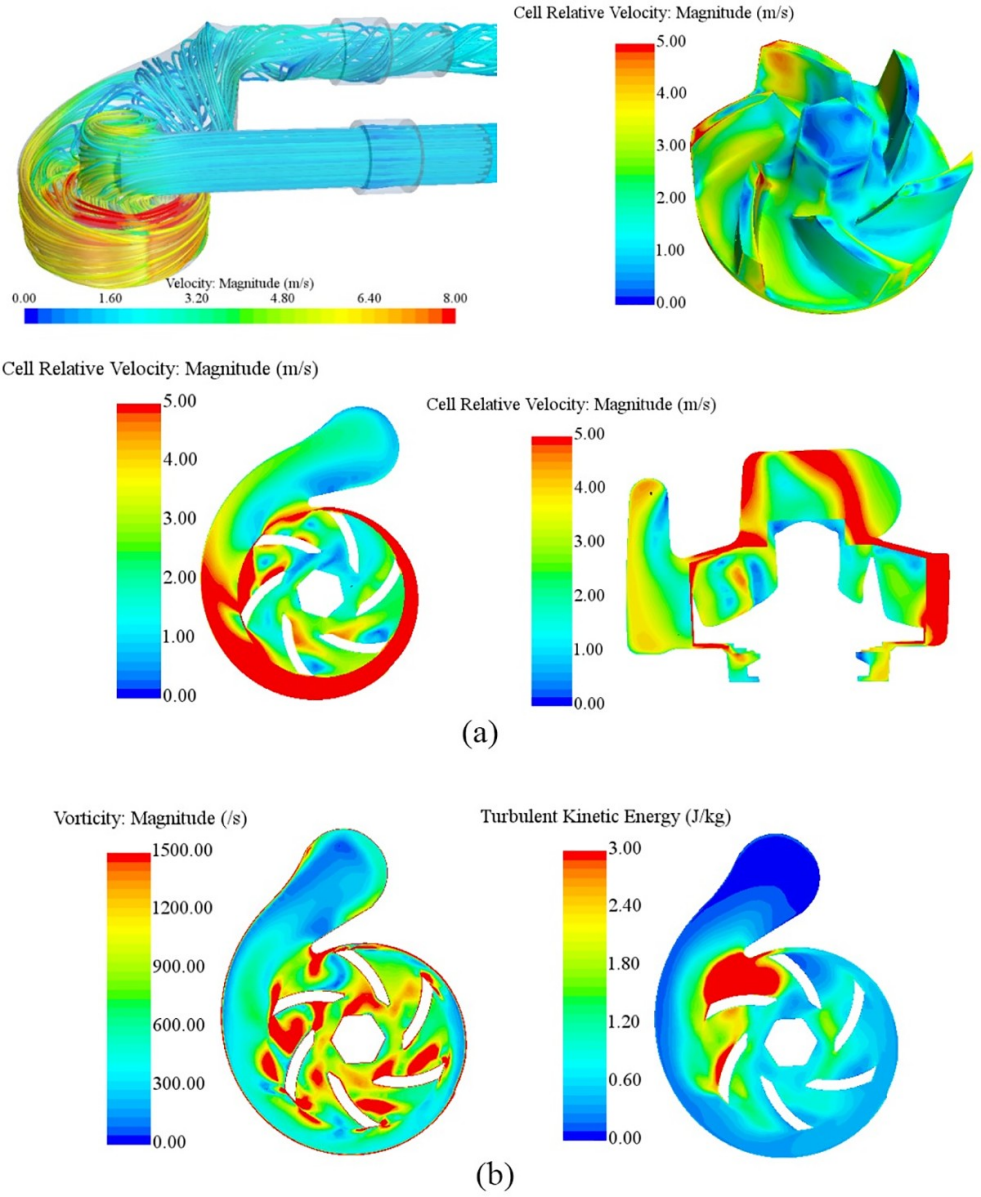
Internal flow field characteristics of original engine cooling water pump: (a) Velocity; (b) Vorticity and turbulent kinetic energy distribution.

Fig 3 showed internal flow field characteristics of engine cooling water pump. The velocity magnitude at the intersection of the impeller outlet and the volute was the largest. The velocity magnitude along the volute became smaller and smaller. The flow field in the volute outlet area tended to become stable gradually, the velocity magnitude decreased due to the diffusion shape resulting in the significant pressure between impeller and volute, and the kinetic energy was converted into pressure energy. The flow field distribution in the pump was affected by the shape and movement form of impeller. The velocity magnitude of fluid at the impeller inlet was small. Being affected by the high speed rotation of impeller, the fluid inside the pump can obtain high dynamic energy converted from the mechanical energy of impeller rotation, resulting that the velocity magnitude of fluid at the impeller outlet became larger. From Fig 3B, it can be seen that turbulent kinetic energy presented an uniform distribution characteristic. Large turbulent kinetic energy area appears in the volute tongue and impeller tip, which indicated that the flow was quite intense. Vorticity was one of the most important physical quantities to describe vortex motion and its distribution characteristics can be considered as an indirect way for representing the area that may have a great chance to form the vortex flow. In the process of impeller rotation, the vorticity value near the impeller tip and volute tongue was quite large, which indicated that those area were most likely to generate vortex flow or flow separation [24–26].

## 4. Hydraulic performance improvement study

### 4.1 Construction of optimization analysis model

**Fig 4:**
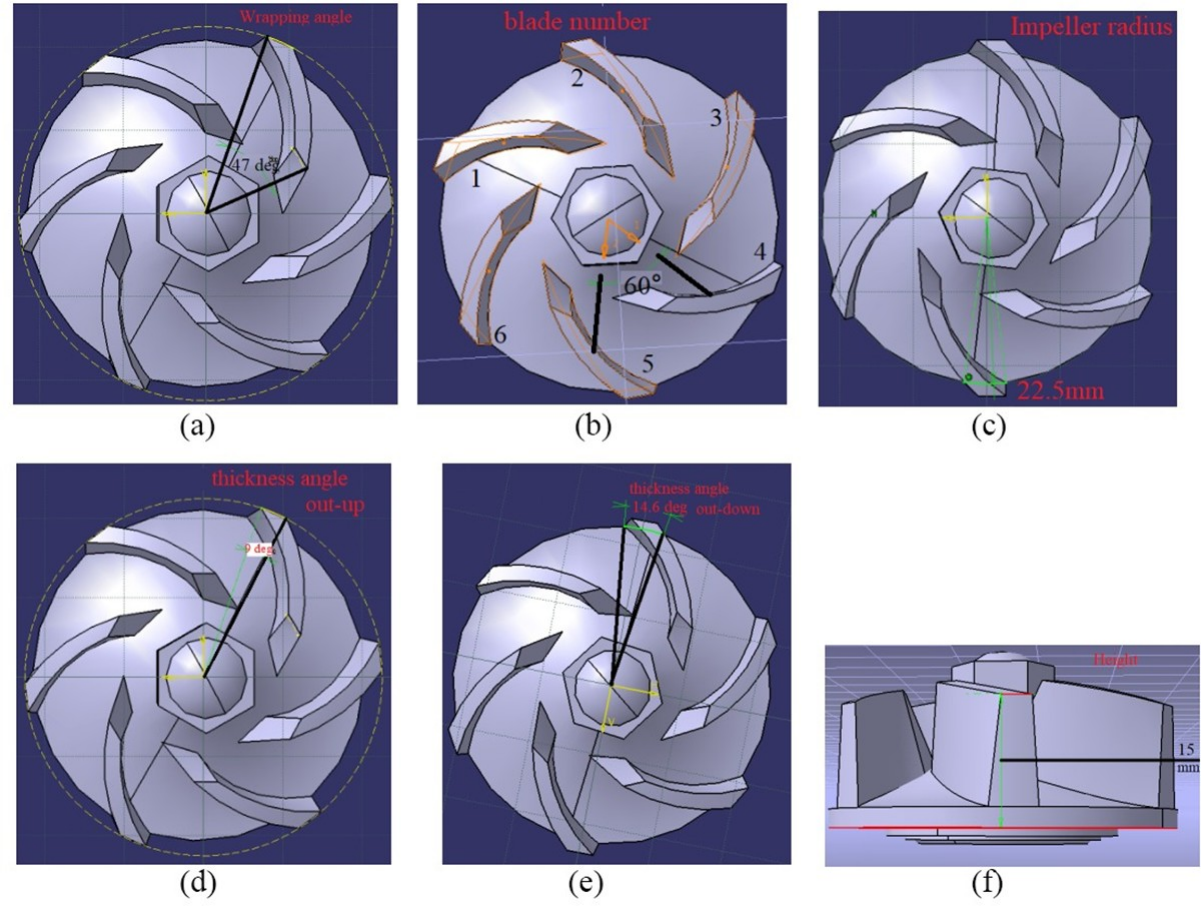
Parametric variables of impeller for hydraulic performance optimization: (a) wrapping angle; (b) blade number; (c) impeller radius; (d) thickness angle out-up; (e) thickness angle out-down; (f) impeller height.

The automatic optimization method by integrating 3D parametric model and CFD simulation software for engineering optimization analysis was widely used in hydraulic performance improvement of pump [27–29]. Therefore, this optimization method was applied in this study by integrating CATIA parametric model and CFD simulation software STAR-CCM+. The parametric model generated by CATIA was used to control the shape of impeller and provide variable structural parameters for hydraulic performance optimization. Fig 4 shows the variable structural parameters of impeller for hydraulic performance optimization. If the impeller height was increased too much, there may be friction or interference between the impeller and the volute operation. Therefore, all the variable structural parameters range of impeller was carefully defined according to the installing limitations in volute, empirical design theory and mutual relationship between parameters for avoiding interference and error generation of 3D model. The parameters of original impeller and variable parameters value range of parametric impeller model were shown in Tables 2 and 3, respectively. The CFD simulation software STAR-CCM+ was used to check hydraulic performance of water pump with the different impeller which was generated by adjusting the variable structural parameters (blade angle, blade number or radius, etc.).

**Table 2:** Impeller parameters of original pump.

Name / symbol	Value	Name	Value
Wrapping angle	*47°*	Upper thickness angle	9°
Blade number	*6*	Lower thickness angle	14.6°
Impeller height	*15 mm*	Impeller radius	*22*.*5 mm*

**Table 3:** Parametric values for impeller.

Name	Symbol	Value
Wrapping angle	A	45°, 50°, 55°, 60°
Blade number	N	4, 5, 6, 7, 8
Impeller height/ mm	H	13, 13.4, 13.8, 14.2, 14.6,15.2
Impeller radius/ mm	R	22,22.1,22.2,22.3,22.4,22.5,22.6,22.7
Thick angleout-up	TA-U	7°, 8°, 9°, 10°, 11°, 12°
Thick angleout-down	TA-D	12°, 13°, 14°, 15°, 16°

**Fig 5:**
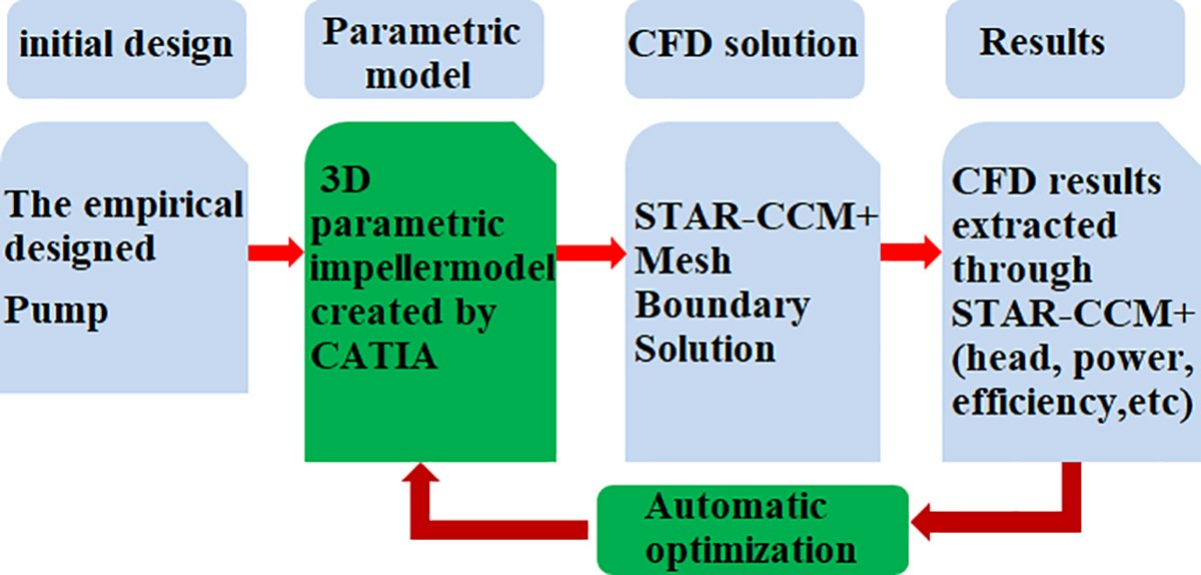
Flowchart of pump hydraulic performance optimization.

Fig 5 showed flowchart of hydraulic performance optimization by integrating 3D parametric model drawing software CATIA and CFD simulation software STAR-CCM+. CFD simulation software was combined with parametric model to realize automatic impeller updating and hydraulic performance evaluation of the updated case model. The surrogate model was applied for the performance optimization. The surrogate model, similar to response surface model, KRG (Kriging model), RBNN (Radial basis neural network), and so on, can reduce the numerical trial to find a good design quickly for achieving the design goal. The best combination of parameters of impeller can be obtained by solving the function with the optimization algorithm programmed by MATLAB or other programming software for linking the parametric model automotive updating with the corresponding CFD simulation. Selecting a good type surrogate model and establishing it was a key step for optimization process. In this work, the Kriging model type of surrogate model was selected for hydraulic performance optimization. The detail description of Kriging model was well described in some researcher’s work [30].

In this research work, the total number of CFD simulation cases was selected as three hundred, and the iteration step of each simulation was two thousand. Each case was calculated by 24 cores of the computer and the completion time for each running case was about thirty minutes. Therefore, the total time of three hundred calculations was about one hundred and fifty hours. In this paper, the volume flow rate of 25 L/min (design point) was selected as operating point to conduct the optimization analysis. STAR-CCM+ was used to build the pump performance calculation model of the selected operating point. The monitoring parameters and its calculation formulas wrote in STAR-CCM+ for hydraulic performance evaluation were shown in Table 4. The head, efficiency, and shaft power of the baseline model in volume flow rate 25 L/min were 3.87 m, 23.09% and 66.7 W, respectively. In the subsequent analysis, the design with higher head and efficiency, lower shaft power can be considered as an optimized design for the engine cooling water pump.

**Table 4:** Monitoring parameters and calculation formulas for hydraulic performance evaluation.

Parameters	Units	Symbol	Formula
Angular velocity	rad/s	*ω*	6.18*(rpm)/60
Pressure difference	Pa	*△P*	*P*_out_*-P*_in_
Mass flowrate	kg/s	*Q*_*m*_	*/*
Head	m	*H*	*△P/ρg*
Torque	N/m	*T*	*/*
Shaft power	W	*P*_*s*_	T*ω
Effective power	W	*P*_*e*_	H*Q_m_*g
Efficiency	%	*η*	100H*Q_m_*g/Tω

### 4.2 Single factor results

**Fig 6:**
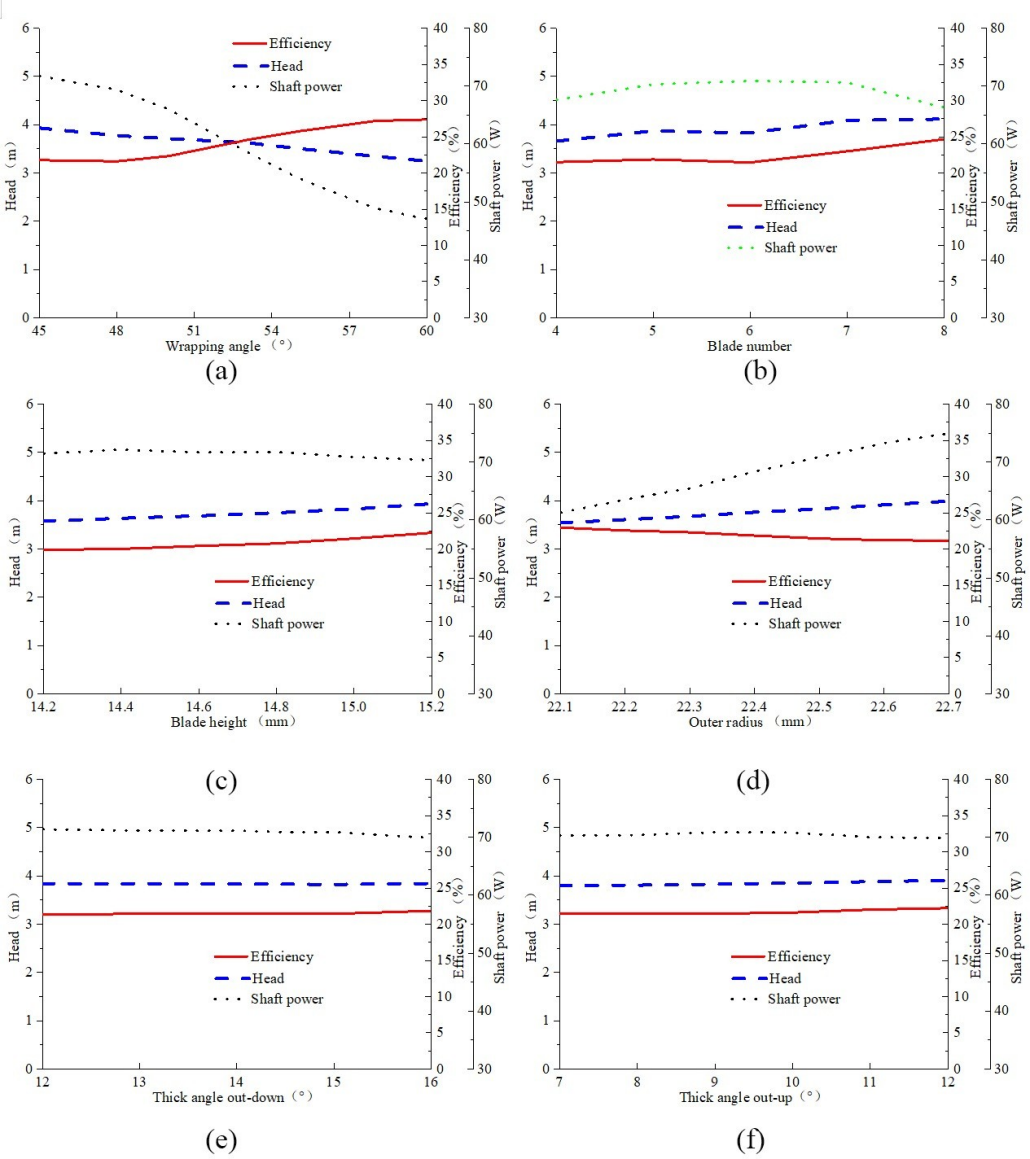
Influence of impeller geometric parameters on pump head and efficiency: (a) wrapping angle; (b) blade number; (c) impeller height; (d) impeller radius; (e) Thick angle out-down; (f) Thick angle out-up.

Fig 6 showed the influence of impeller geometric parameters on pump head and efficiency. The six geometric parameters (wrapping angle, blade number, impeller height, impeller radius, thick angle out-up and thick angle out-down) were chosen for investigating the single factor influence on pump hydraulic performance. As shown in Fig 6, pump head decreased with the increase of wrapping angle, and hydraulic efficiency increased with the increase of wrapping angle (Fig 6A). The reason was that the shaft power reduce significantly with the increase of wrapping angle. The head and efficiency firstly increased with the increment of blade number and then it had the tendency to decrease (Fig 6B). Therefore, as for a certain pump, there was always existing an optimum blade number for obtaining a better hydraulic performance. The pump head increased with the increase of impeller height, while there was existing an optimum impeller height for obtaining a better pump efficiency (Fig 6C). Pump head and pump efficiency increased with the increment of impeller radius, as for the thickness angle, it seemed that the thickness angle had no obvious effect on the pump performance characteristics (Fig 6D–6F). Reason for the efficiency increasing or decreasing was due to the reduce or increase of shaft power respectively.

Table 5 showed the correlation analysis between two parameters. For example, the correlation value between the shaft power and efficiency was -0.24, it indicated that when the shaft power was increased, the efficiency was decreased. As for the impeller structural parameters on pump performance, in the row of wrapping angle, the correlation between wrapping angle and power, efficiency, head were -0.71, 0.63, -0.36, respectively, indicating that the head was decreased when the wrapping angle was increased. It can be seen from Table 5, the performance parameters were affected by the structural parameters, and there was a restraining relationship among those structural parameters, which made it very difficult to find the optimal scheme through the single factor analysis method.

**Table 5:** Correlation analysis between two parameters.

parameters	Correlation								
	power	efficiency	head	wrapping angle	blade number	impeller height	outer radius	Thick angleout-down	Thick angleout-up
power	/	-0.24	0.82	-0.71	-0.33	-0.10	0.46	-0.06	0.15
efficiency	/	/	0.28	0.63	0.48	0.27	-0.29	0.23	-0.18
head	/	/	/	-0.36	0.11	0.20	0.41	0.07	0.07
wrapping angle	/	/	/	/	0.24	0.17	-0.15	0.15	-0.14
blade number	/	/	/	/	/	0.22	-0.08	0.13	-0.10
impeller height	/	/	/	/	/	/	0.07	0.05	0.02
outer radius	/	/	/	/	/	/	/	0.07	0.03
Thickangle out	/	/	/	/	/	/	/	/	-0.04
Thickangleout-up	/	/	/	/	/	/	/	/	/

### 4.3 Optimization results

#### 4.3.1 Model prediction accuracy

**Fig 7:**
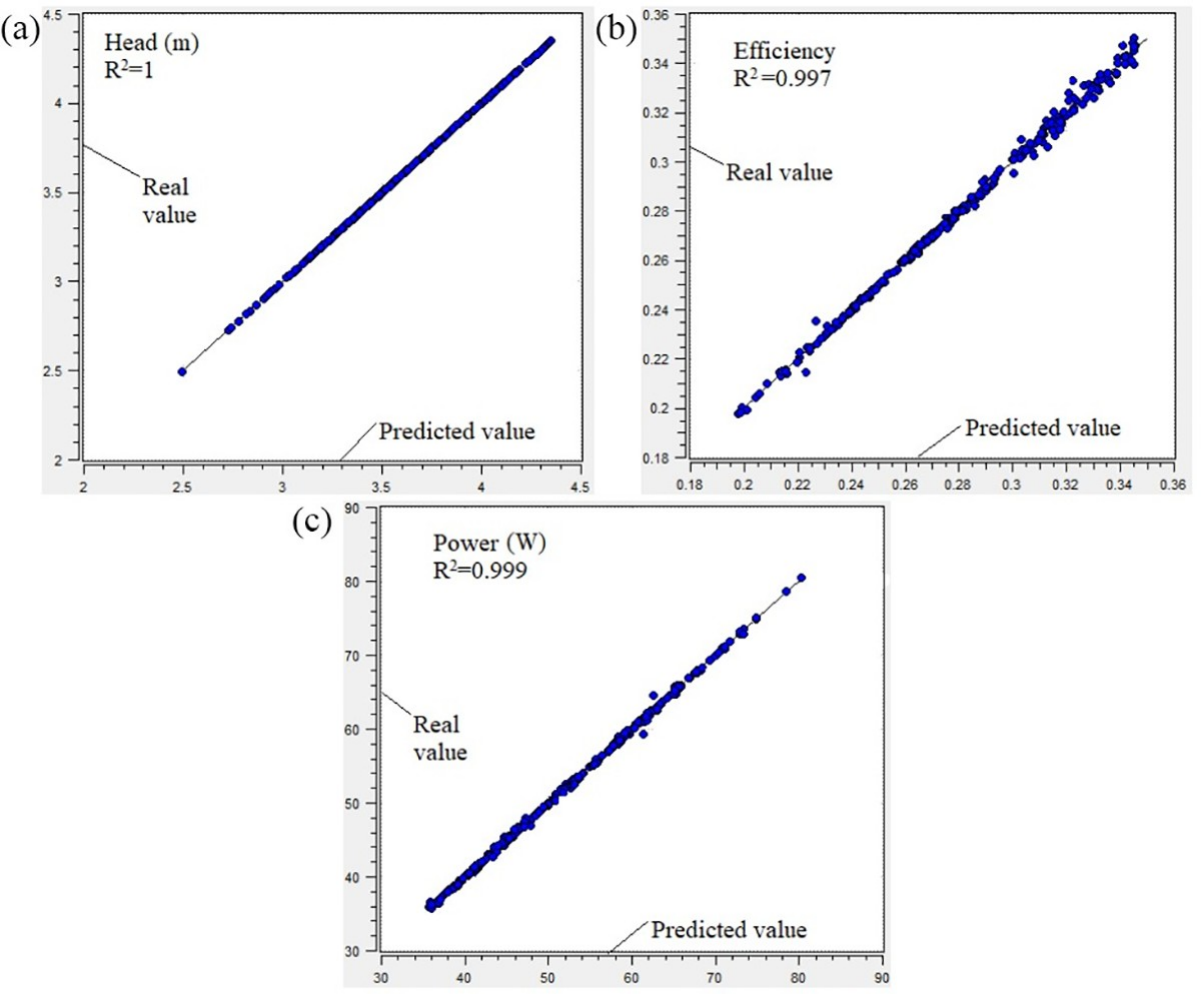
Prediction accuracy of optimization model: (a) head; (b) efficiency; (c) power.

Evaluating the accuracy of the selected surrogate model for performance optimization was necessary. Thus, the error analysis method of R-square was used to evaluate the prediction accuracy of optimization model. If the R-square value was more than 0.9, then the optimization model had good prediction accuracy. Fig 7 showed that the R-square values were 1, 0.997, 0.999 for the prediction of head, efficiency, and shaft power respectively, indicating the model prediction accuracy is quite well. Therefore, the optimized designs obtained after optimization analysis completed were effective and reliable.

#### 4.3.2 Optimized designs

The structural parameters had obvious influence on hydraulic performance of pump. Previous studies showed pump head increased with the increase of impeller blade number, while there was existing an optimum impeller blade number for obtaining a better efficiency at a certain operating point [31]. The pump head and efficiency for a pump increased with the decrease of impeller outlet angle [32]. Some other parameters (impeller outer radius, impeller height) were also related to hydraulic performance. Therefore, compared to manual design optimization, automatic optimization by the integration of parametric model and hydraulic performance simulation was a fast and reliable method to obtain optimized designs.

Table 6 showed four potential optimized designs for engine cooling water pump model at the designed volume flow rate of 25 L/min. Optimized design No.1 have the largest efficiency, optimized design No.2 have the largest head, optimized design No.3 have the best comprehensive effect of head, efficiency and shaft power improvement, and optimized design No.4 have some improvement with the circumstance that impeller outer diameter remains unchanged. The head and efficiency of those four optimized designs were better than that of the original engine cooling water pump. For the optimized design No.1, the efficiency from the origin state of 23.1% to the current state of 29.1%, 6% enhanced. For the optimized design No.2, the pump head from the origin state of 3.87 m to the current state of 4.22 m, 0.35 m enhanced and the increment ratio was 9%.

**Table 6:** Optimization results of engine cooling water pump.

Name	A	N	H	R	TA-D	TA-U	Head	Efficiency	Shaft power
	(°)	-	(mm)	(mm)	(mm)	(°)	(m)	(%)	(W)
Origin	47	6	15	22.5	14.6	9	3.87	23.1	66.7
Opt1	57	8	15.2	22.7	15.5	11	3.89	29.1	53.3
Opt2	52	8	15.2	22.7	15.5	11	4.22	27.1	61.9
Opt3	54	8	15.2	22.6	16	10	3.93	28.2	55.3
Opt4	48	8	15.2	22.5	15.5	12	4.17	26.4	62.7

**Fig 8:**
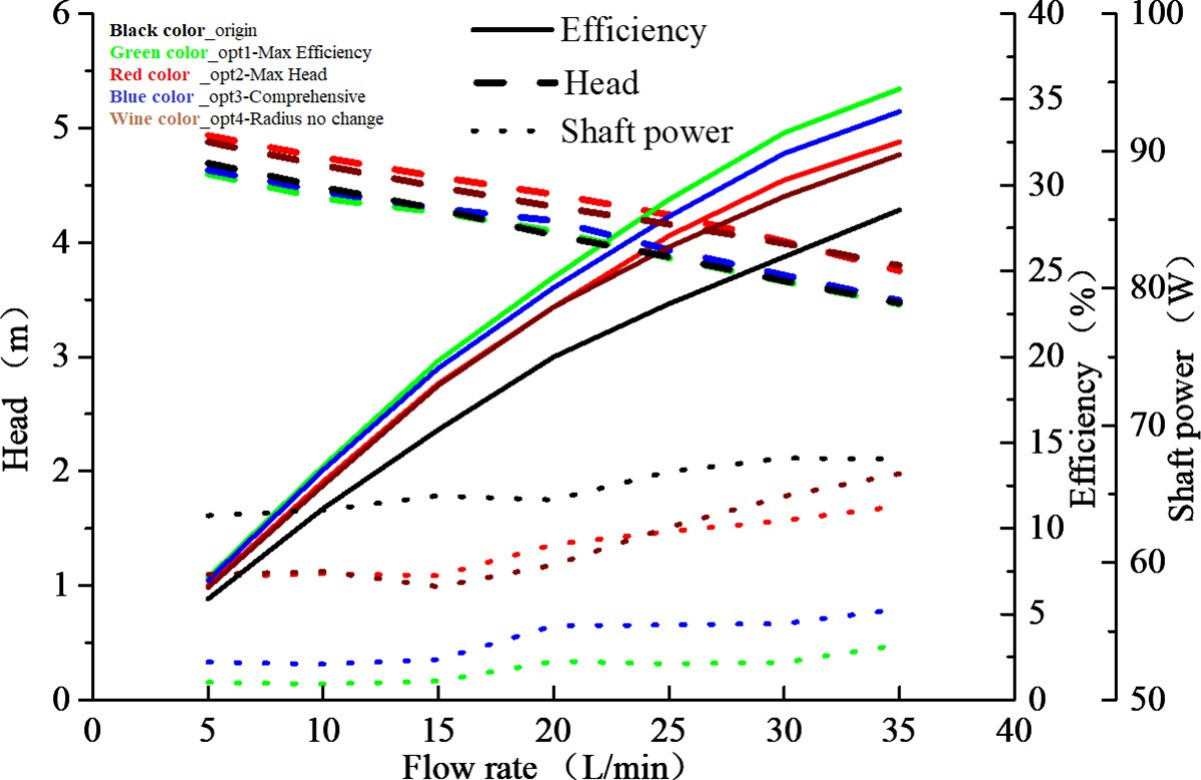
Hydraulic performance curves.

Fig 8 showed hydraulic performance comparison curves of engine cooling water pump. After optimization, the head and efficiency of optimized pump were improved and the shaft power of optimized pump was reduced. Optimized design No.1 and optimized design No.3 can be considered as good designs for the reason that this two designs can obtain a good hydraulic performance with higher efficiency and lower shaft power. For making comparison between the optimized design No.1 and optimized design No.3, the pump head was a little higher than that of the optimized design No.1. For comprehensive improving effect, the optimized design No.3 can be considered as the better design. So, in experimental validation sections, the optimized design No.3 (Opt 3) was selected as one of the optimized design for checking the performance improvement of engine cooling water pump and verifying the effectiveness and reliability of hydraulic performance optimization analysis.

### 4.4 Experimental validation

**Fig 9:**
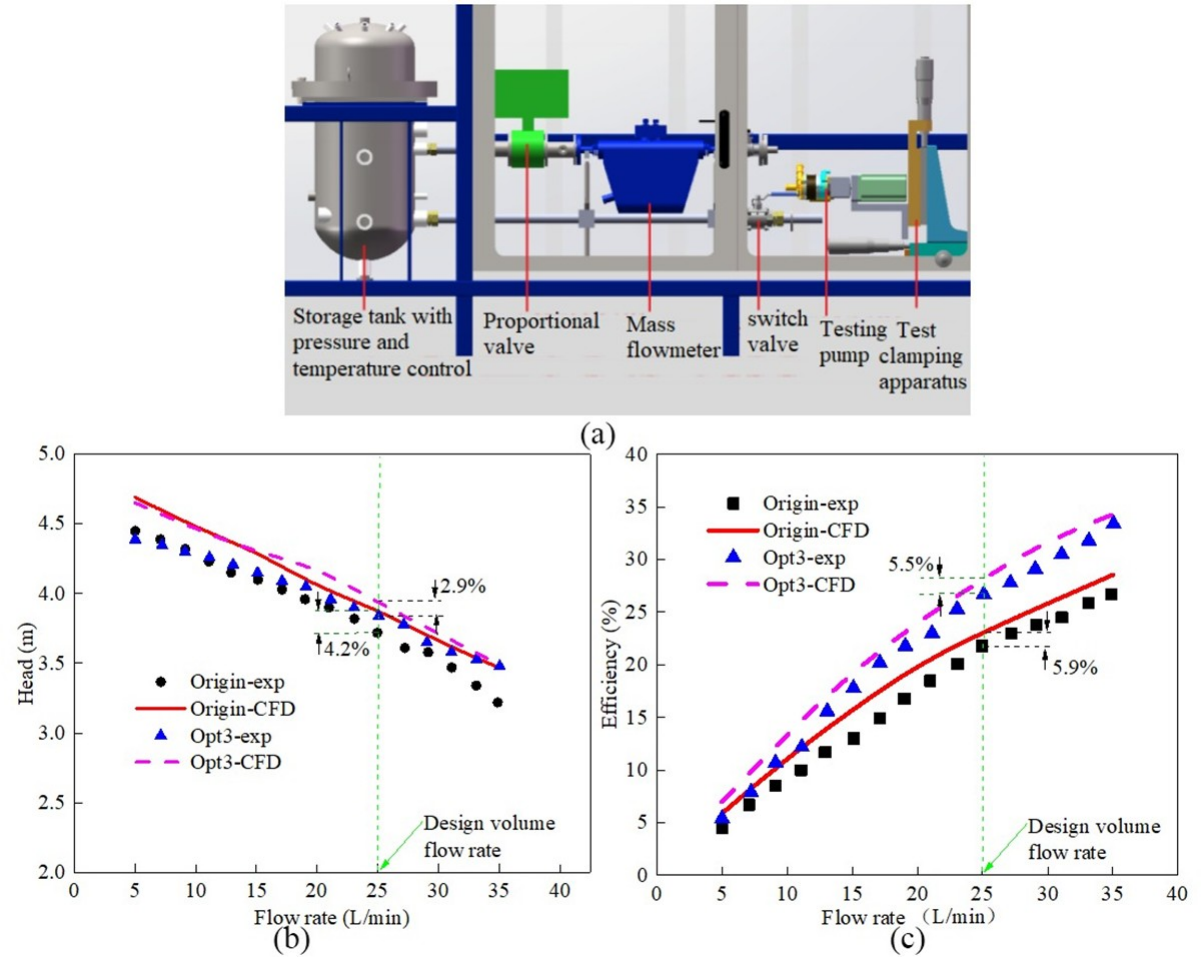
Experimental validation analysis: (a) Testing apparatus; (b) Head curves; (c) Efficiency curves.

Fig 9 showed the hydraulic performance testing apparatus (Fig 9A) and the experimental validation results for engine cooling water pump (Fig 9B and 9C). The test device mainly included mass flowmeter, liquid storage tank, proportional valve, pressure sensor, testing pump, testing clamping apparatus, driving motor and other pipe components. The measuring range of pressure sensor was 0 kPa~500 kPa and the error level was 0.1%. The measuring range of Coriolis flowmeter was 2 L/min~140 L/min and the error level was 0.1%. The measuring range of shaft torque meter and rotational speed meter was 0~2 N·m and 0~12000 rpm respectively, and the error level for this two meters were 0.2%. The hydraulic performance test of engine cooling pump was carried out in accordance with the China national standard requirements for hydraulic performance test of automobile engine cooling water pump [33, 34]. The testing pump was connected to the test apparatus through special self-designed testing clamping apparatus, and the driving motor drove the pump to work at a certain rotational speed. In the whole test process, the mass flowrate of the testing liquid (pure water) was adjusted by proportional valve and measured by the mass flowmeter. At a certain mass flow rate, the pressure sensor installed at the pump inlet and pump outlet for measuring pressure before pump inlet and pressure after pump outlet was obtained automatically to get the pressure difference. The test condition change and corresponding hydraulic performance data record were all automatically completed through the test device console. The performance curves tested by measurements was automatically processed and analyzed by pump hydraulic performance data processing program integrated in the test system, and the corresponding pump performance curves (flowrate vs head curve, flowrate vs efficiency curve) were generated.

The experimental validation results (Fig 9B and 9C) showed that the CFD and experimental hydraulic efficiencies and head illustrated a good agreement. For original water pump model at volume flowrate condition of 25 L/min, compared to the experimental measurements, the pump head and hydraulic efficiency results from CFD modeling showed 4.2% difference and 5.9% difference, respectively. For optimized pump design No.3 at volume flowrate condition of 25 L/min, the relative error between the simulated and experimental values were 2.9% for head and 5.5% for efficiency. As a whole, the numerical method and optimization method adopted in this work is reasonable and effective for hydraulic performance prediction and optimization for engine cooling water pump. The research results can give a guiding reference for design engineer to design engine cooling water pump with high hydraulic performance.

## 5. Conclusions

In this work, the hydraulic performance evaluation and optimization analysis for an engine cooling water pump was investigated by CFD simulation. The optimization method by integrating parametric model and CFD simulation software was adopted to seek the optimized design for engine cooling water pump. The main conclusions drawn were given as follows:

1. The head, efficiency, and shaft power of original engine cooling water pump at designed volume flow rate 25 L/min were 3.87 m, 23.09% and 66.7 W, respectively and compared to the experimental measurements, the pump head and hydraulic efficiency results from CFD modeling showed 4.2% difference and 5.9% difference, respectively.
2. Through hydraulic performance optimization, four optimized design were put forward. For the optimized design No.1, the efficiency from the origin state of 23.1% to the current state of 29.1%, 6% enhanced. For the optimized design No.2, the pump head from the origin state of 3.87 m to the current state of 4.22 m, 0.35 m enhanced and the increment ratio was 9%. For comprehensive improving effect, the optimized design No.3 can be considered as the better design.
3. The CFD and experimental hydraulic efficiencies and head illustrated a good agreement. For the optimized pump design No.3 at volume flowrate condition of 25 L/min, relative error between the simulated and experimental values were 2.9% for head and 5.5% for efficiency. The simulation analysis data and optimization conclusion can provide the simulation data support and theoretical basis for the structural design and optimization of engine cooling water pump.

## Supporting information

S1 File (XLSX)
